# UNDERSTANDING THE ROLE OF EDUCATION IN COGNITIVE FUNCTIONING AMONG OLDER BRAZILIANS

**DOI:** 10.1093/geroni/igae098.0276

**Published:** 2024-12-31

**Authors:** Mateo Farina

**Affiliations:** University of Texas at Austin, Austin, Texas, United States

## Abstract

Education has been linked to cognitive functioning in older adulthood across time and place. However, how education is linked to later life cognitive functioning may differ across country contexts. The role of education has been widely debated with evidence for both direct and indirect pathways (i.e. occupation, health behaviors, etc.). However, much of the research has been limited in low- and middle-income countries that, in general, have lower levels of education and greater levels of social inequity. As one of the fastest-aging countries that has seen substantial improvements in education levels over the last 60 years but with significant social inequality, Brazil provides a prime example to understand how education as an exposure or the sequelae of pathways from education may impact cognitive health in older adulthood. This study uses nationally representative data from the ELSI-Brazil Study (50+) to examine the association of education with older adult cognitive functioning (global and domain-specific). I used a series of linear regression models to assess the sensitivity of the association of education with cognitive functioning after adjustments for health behaviors, health conditions, and wealth. I found that education was positively associated with cognitive functioning; for the total population, it remained relatively unchanged with downstream adulthood factors. Nevertheless, among those with more than 8 years of education, differences were attenuated by downstream factors. This suggests that education as an exposure may drive future trends in cognitive health in countries with low levels of overall education.

